# A Novel Particle Filter Based on One-Step Smoothing for Nonlinear Systems with Random One-Step Delay and Missing Measurements

**DOI:** 10.3390/s25020318

**Published:** 2025-01-08

**Authors:** Zhenrong Yang, Xing Zhang, Wenqian Xiang, Xiaohui Lin

**Affiliations:** 1 School of Mathematics and Information Science, Guangxi University, Nanning 530004, China; zhenrongy111@163.com (Z.Y.); xiangwenqian2024@163.com (W.X.); linxh0304@163.com (X.L.); 2 Center for Applied Mathematics of Guangxi (Guangxi University), Guangxi University, Nanning 530004, China

**Keywords:** particle filter, nonlinear filter, one-step delay, missing measurements, importance function, one-step smoothing

## Abstract

In this paper, a novel particle filter based on one-step smoothing is proposed for nonlinear systems with random one-step delay and missing measurements. Such problems are commonly encountered in networked control systems, where random one-step delay and missing measurements significantly increase the difficulty of dynamic state estimation. The particle filter is a nonlinear filtering method based on sequential Monte Carlo sampling. It shows excellent state estimation performance when dealing with nonlinear and non-Gaussian dynamic systems. However, the particle filter has certain limitations in complex dynamic scenarios, with particle degradation being the most typical issue, which can significantly reduce estimation accuracy. To address particle degradation, the proposed particle filter iteratively incorporates current measurement information derived from sensors into the prior distribution to construct a new importance function. This approach can limit particle degeneracy and improve the efficiency of importance sampling in the bootstrap particle filter. Simulation experiments demonstrate that the proposed particle filter effectively limits particle degradation and improves estimation accuracy compared to the existing bootstrap particle filter for nonlinear systems with random one-step delay and missing measurements.

## 1. Introduction

In modern scientific tools and techniques, sensors often provide data with noise [1]. This noise can arise from sensor inaccuracies or external disturbances. Similarly, in non-engineering fields, measurement and experimental data are also affected by noise [2]. To determine the hidden state of a system or process from noisy data, a common mathematical approach is state estimation. This process is often performed recursively and is referred to as filtering [3]. Filtering algorithms are widely applied in various fields, including target tracking [4], biomedical modeling [5], industrial diagnostics [6], and financial derivatives price prediction [7]. Currently, the most widely used type of state estimation filter is the Bayesian filter. This filter uses the Bayesian framework and provides a state estimation method by utilizing a priori and a posteriori probability density functions (PDFs).

The well-known Kalman filter (KF) [8] is an optimal linear Bayesian filter. However, in practical applications, many systems are not simple linear systems but have complex nonlinear properties [9,10], making the traditional KF inapplicable. To solve this problem, several improved KFs have been proposed. For example, Kalman-like filters include the extended Kalman filter (EKF) [11], unscented Kalman filter (UKF) [12], cubature Kalman filter (CKF) [13], and high-degree cubature Kalman filter (HCKF) [14]. These methods are widely used due to their computational efficiency. However, these filters rely on Gaussian assumptions, limiting their applicability to complex nonlinear systems. To address this difficulty, the particle filter (PF) has emerged [15,16]. The PF uses a set of weighted random samples (also called particles) to estimate the posterior distribution according to the Monte Carlo sampling principle. It does not rely on Gaussian assumptions, does not have any restrictions on the system equations and noise, and has good estimation accuracy in solving complex nonlinear non-Gaussian systems [17,18]. However, in practice, the PF often suffers from particle degeneration and can be computationally intensive, as a large number of particles are required to achieve improved estimation accuracy [19]. Based on sequential importance sampling theory, the particles are sampled sequentially from an importance function. The weight of each sample is calculated by the proportion between the posterior distribution and the importance function. Particle degradation, also known as weight degradation, is a common problem in the PF. The primary reason for particle degradation is that the importance function significantly differs from the true posterior distribution. This mismatch causes particles to be sampled from low-probability regions of the posterior distribution, which leads to an uneven distribution of particle weights. Over the course of iterations, this imbalance causes the weight variance to increase progressively. As a result, most particles end up with very low weights, while only a few particles maintain high weights. During the resampling process, higher-weighted particles are repeatedly selected, while lower-weighted particles are discarded. This results in most of the remaining particles being concentrated among a minority with high weights, causing particle degradation and unreliable estimation results.

The performance of the PF depends heavily on the choice of importance function. A smaller difference between the importance function and the posterior distribution results in particles drawn from the importance function that more closely approximate the posterior [20,21]. Therefore, choosing a suitable importance function can make it closer to the posterior distribution, which can effectively limit the particle degradation, improve the sampling efficiency and reduce the computational cost. This has lead to recent studies on PFs that focus on designing new importance functions. The bootstrap particle filter (BPF) [22] is a classical PF that uses the priori distribution as the importance function. However, the importance function in the BPF does not contain observation information, which may lead to particle degradation. An effective way to limit particle degradation is to directly choose the posterior distribution as the importance function. However, for nonlinear systems, the posterior distribution is usually difficult to sample directly. Therefore, some studies on importance functions have focused on approximating the density function of the posterior distribution. One common approach uses the density function of a Gaussian optimal filter as the importance function, i.e., the Gaussian PF [23,24]. Another research direction is to construct density functions using the latest measurement information as the importance function. Examples include the auxiliary PF [25], the genetic PF [26], and the intelligent PF [27]. These PFs propose an efficient way to select the importance function to limit particle degeneration. However, all these PFs are for nonlinear systems, assuming that the observed data always contain continuous useful information.

In many practical applications, random time delays and missing measurements (MMs) inevitably occur, reducing the filtering accuracy of the algorithm [28,29]. For example, in real-time wireless control systems, ultra-reliable low-latency communication (URLLC) is essential to ensure stable communication between remote controllers, sensors, and controlled objects. However, transmission delays and MMs from sensors can significantly affect the control performance [30]. In addition, in networked control systems, the choice of controller location is critical to mitigate the effects of missing measurements in the network. However, this optimization problem is very complex and difficult to solve due to MMs and incomplete information [31]. Thus, in this paper, we focus on the nonlinear systems with random one-step delay and MMs. The random one-step delay and MMs are modeled by two random variables, both obeying the Bernoulli distribution. As with traditional nonlinear models, both the state and measurement functions are nonlinear with the system and measurement noise being additive Gaussian random variables. However, due to the introduction of random one-step delay and MMs, the measurement equation incorporates two multiplicative noises following Bernoulli distributions, which significantly increase the model complexity.

Random time delays and MMs have garnered significant research attention, resulting in numerous published studies [32,33]. Recently, the optimal filtering problem for discrete stochastic systems with finite-step autocorrelation process noise, random one-step sensor delays and MMs has been studied using the state generalization method and the principle of minimum mean square error [34]. In [35], an optimal linear filter is designed for discrete-time systems with measurement delays and MMs. In [36], a Gaussian filter is applied to address simultaneously occurring delays and MMs. However, few studies have explored the PF problem for random one-step delay and MMs. Currently, we only find that [37] uses the BPF applied to nonlinear systems with random one-step delays and MMs. However, the BPF selects the prior distribution as the importance function. Since this importance function does not incorporate observational information, it may result in particle degradation.

In summary, in order to limit the particle degeneracy in the BPF, we propose a novel PF based on one-step smoothing for nonlinear systems with random one-step delay and MMs. Unlike the importance function of the BPF, which does not contain any information from the current measurements, this newly proposed filter iteratively uses a new one-step particle smoother by some way introducing information from the current measurements into the importance function. This iterative approach to one-step particle smoothing aims to use the current measurements to improve the importance function, making it closer to the posterior distribution.

The main contributions of this paper are as follows: (1) proposing one-step smoothing by incorporating current measurement information into the importance function to construct a new importance function and an explicit expression for the corresponding weights; (2) designing a novel PF algorithm for nonlinear systems with random one-step delay and MMs, effectively limiting particle degeneracy and improving estimation accuracy.

This paper is organized as follows. In Section 2, the nonlinear systems with random one-step delay and MMs are presented. In Section 3, the one-step particle smoother is introduced. In Section 4, by incorporating the current measurement information, we propose an iterative scheme based on one-step smoothing to construct a more optimal importance function and an explicit expression for the corresponding weights, thereby providing a novel PF algorithm for the system under discussion. Section 5 presents simulations for demonstration, and Section 6 provides the conclusions.

## 2. Nonlinear Systems with Random One-Step Delay and Missing Measurements

Consider dynamic systems with state space formalisms that can be described in the following:

(1) $$\begin{array}{cc} x_{t+1} & =f_{t}\left(x_{t}\right)+w_{t} \end{array}$$

(2) $$\begin{array}{cc} z_{t} & =h_{t}\left(x_{t}\right)+v_{t} \end{array}$$

Here, $x_{t}\in R^{n}$ and $z_{t}\in R^{m}$ represent states and ideal measurements (i.e., no delays and missing), respectively. f(·) and h(·) are known state and measurement functions, respectively, both of which are nonlinear. $w_{t}\in R^{n}$ and $v_{t}\in R^{m}$ are system noise and measurement noise, respectively. They are Gaussian white noise independent of each other. Let $w_{t}∼N\left(0,Q_{t}\right)$, $v_{t}∼N\left(0,R_{t}\right)$.

However, in practical applications such as networked control systems, sensors often generate measurements with random one-step delays or missing data due to network congestion or communication failures. As a result, the ideal measurement $z_{t}$ may become unavailable. Considering these challenges, the measurement $y_{t}$, which is generated by the sensors, can be modeled as

(3) $$\begin{array}{cc} y_{t} & =\lambda_{t}z_{t}+\left(1-\lambda_{t}\right)\xi_{t}z_{t-1}+\left(1-\lambda_{t}\right)\left(1-\xi_{t}\right)v_{t} \end{array}$$

The random variables $\lambda_{t}$ and $\xi_{t}$, representing random one-step delays and MMs, follow a Bernoulli distribution with the following statistical characteristics:

$$\begin{array}{cc} P(\lambda_{t}=1) & =E\{\lambda_{t}\}=a,\\ P(\lambda_{t}=0) & =1-E\{\lambda_{t}\}=1-a,\\ P(\xi_{t}=1) & =E\{\xi_{t}\}=b,\\ P(\xi_{t}=0) & =1-E\{\xi_{t}\}=1-b, \end{array}$$

where a and b are known constants in the interval [0, 1].

**Remark** **1.** *For the model* (3)*, the following three situations occur.:*

- *When $\lambda_{t}=1$, $y_{t}=z_{t}$, it means that the sensor successfully obtains useful information.*
- *When $\lambda_{t}=0$ and $\xi_{t}=1$, $y_{t}=z_{t-1}$, it means that the sensor experiences a one-step delay.*
- *When $\lambda_{t}=0$ and $\xi_{t}=0$, $y_{t}=v_{t}$, it means that the sensor measurement is missing.*

**Figure 1:**
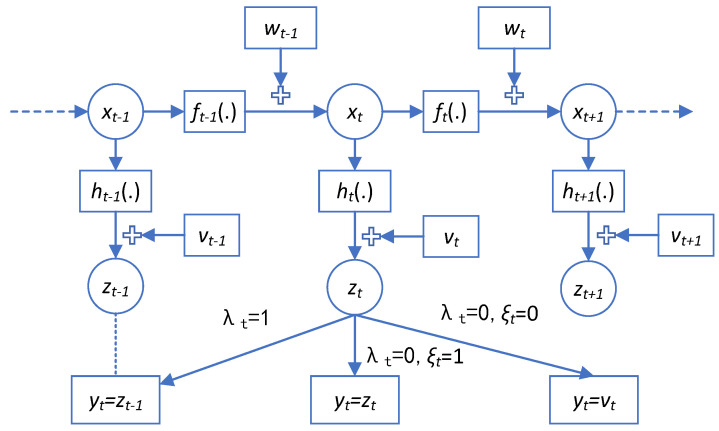
Random one-step delay and MMs.

To better illustrate the dynamic process of the system described in (1)–(3), a schematic representation is provided in Figure 1.

As a convenience, let $x_{0:t}=\left\{x_{0},\ldots ,x_{t}\right\}$ and $y_{0:t}=\left\{y_{0},\ldots ,y_{t}\right\}$. To simplify the subsequent proof, we adopt the following two assumptions, as in the reference [37]:

**Assumption** **1.** *The state $x_{t}$ follows a first-order Markov process:*

$$p\left(x_{t}|x_{0:t-1},y_{0:t}\right)=p\left(x_{t}|x_{t-1}\right).$$

**Assumption** **2.** *The sensor output $y_{t}$ and states $x_{t},x_{t-1}$ are related by*

$$p\left(y_{t}|x_{0:t},y_{0:t-1}\right)=p\left(y_{t}|x_{t},x_{t-1}\right).$$

## 3. One-Step Particle Smoother

### 3.1. Weighted Sample Approximation with Importance Sampling Theory

Within the framework of SIS theory, the PF has become a powerful tool for approximating posterior distributions by employing a set of weighted samples. To provide a clearer understanding of this process, this section revisits the concept of importance sampling and its role in weighted sample approximation. The concept of “approximately weighted samples” is used in [38] to incorporate PF methods into the unified framework of SIS. To verify that a set of weighted samples approximates a target distribution, we provide the following proposition, which is based on the viewpoint of [39].

**Proposition** **1.** *For a set of weighted random samples $\left\{x^{\left(i\right)},W^{\left(i\right)}\right\}$, if it approximately represents a target distribution p, then for any measurable set B, the following equation holds:*

$$\lim_{N\to \infty }\frac{\sum_{i=1}^{N}\chi_{B}\left(x^{\left(i\right)}\right)W^{\left(i\right)}}{\sum_{i=1}^{N}W^{\left(i\right)}}=E_{p}\left[\chi_{B}\left(X\right)\right],$$

*where $\chi_{B}\left(\cdot \right)$ is the indicator function of the set B.*

The detailed proof of Proposition 1 can be found in [39].

Based on Proposition 1, we now consider the use of importance sampling to obtain a weighted sample. This leads to the following proposition, which establishes the relationship between two distributions p(x) and π(x), and the corresponding importance weights.

**Proposition** **2.** *Given two distributions p(x) and π(x), suppose that the samples $x^{\left(i\right)}$ are drawn from the importance function π(x), and the weights $W^{\left(i\right)}$ are defined by:*

$$W^{\left(i\right)}=\frac{p(x^{\left(i\right)})}{\pi (x^{\left(i\right)})},\;i=1,2,\ldots ,N.$$

*Then, the set of weighted samples $\left(x^{\left(i\right)},W^{\left(i\right)}\right)$ approximately represents the target distribution p(x). Specifically, for any measurable set B, the following equation holds:*

$$\lim_{N\to \infty }\frac{\sum_{i=1}^{N}\chi_{B}\left(x^{\left(i\right)}\right)W^{\left(i\right)}}{\sum_{i=1}^{N}W^{\left(i\right)}}=E_{p}\left[\chi_{B}\left(X\right)\right]$$

**Proof.**  Based on the law of large numbers, for any measurable set *B*, we have

(4) $$\lim_{N\to \infty }\frac{\sum_{i=1}^{N}\chi_{B}\left(x^{\left(i\right)}\right)W^{\left(i\right)}}{\sum_{i=1}^{N}W^{\left(i\right)}}=\lim_{N\to \infty }\frac{\frac{1}{N}\sum_{i=1}^{N}\chi_{B}\left(x^{\left(i\right)}\right)W^{\left(i\right)}}{\frac{1}{N}\sum_{i=1}^{N}W^{\left(i\right)}}=\frac{E_{\pi }\left[\chi_{B}\left(X\right)W\left(X\right)\right]}{E_{\pi }\left[W\left(X\right)\right]}$$

Based on the weight $W\left(x\right)=\frac{p(x)}{\pi (x)}$. For the numerator of Equation (4),

$$E_{\pi }\left[\chi_{B}\left(X\right)W\left(X\right)\right]=\int \chi_{B}\left(x\right)\frac{p(x)}{\pi (x)}\pi \left(x\right)dx=\int \chi_{B}\left(x\right)p\left(x\right)dx.$$

For the denominator,

$$E_{\pi }\left[W\left(X\right)\right]=\int \frac{p(x)}{\pi (x)}\pi \left(x\right)dx=\int p\left(x\right)dx=1.$$

Thus,

$$\frac{E_{\pi }\left[\chi_{B}\left(X\right)W\left(X\right)\right]}{E_{\pi }\left[W\left(X\right)\right]}=\frac{\int \chi_{B}\left(x\right)p\left(x\right)dx}{1}=\int \chi_{B}\left(x\right)p\left(x\right)dx=E_{p}\left[\chi_{B}\left(X\right)\right].$$

Therefore,

$$\lim_{N\to \infty }\frac{\sum_{i=1}^{N}\chi_{B}\left(x^{\left(i\right)}\right)W^{\left(i\right)}}{\sum_{i=1}^{N}W^{\left(i\right)}}=E_{p}\left[\chi_{B}\left(X\right)\right].$$

This proves that the weighted samples $\left\{x^{\left(i\right)},W^{\left(i\right)}\right\}$ approximately represent the target distribution p(x). This proof is complete.    □

According to Proposition 2, the target distribution p(x) can be approximately represented by a set of weighted samples. This representation is based on the principle of importance sampling, where samples draw from the proposal distribution π(x).

### 3.2. One-Step Smoothing for Weighted Samples

It is well known that the goal of filtering is to recursively obtain the conditional probability density $p(x_{t}|y_{0:t})$ from $p(x_{t-1}|y_{0:t-1})$. In the PF, this process is approximated by representing the posterior distributions $p(x_{t}|y_{0:t})$ with weighted samples, i.e.,

(5) $$p\left(x_{t}|y_{0:t}\right)=\sum_{i=1}^{N}{\tilde{W}}_{t}^{\left(i\right)}\delta \left(x_{t}-x_{t}^{\left(i\right)}\right),$$

where δ denotes the Dirac measure, and the sample $x_{t}^{\left(i\right)}$ is drawn from the importance function $\pi (x_{t}|y_{0:t})$,

(6) $$W_{t}^{\left(i\right)}=\frac{p(x_{t}|y_{0:t})}{\pi (x_{t}|y_{0:t})}$$

is the importance weight, and

(7) $${\tilde{W}}_{t}^{\left(i\right)}=\frac{W_{t}^{\left(i\right)}}{\sum_{i=1}^{N}W_{t}^{\left(i\right)}}$$

is the normalized weight. The samples $x_{t}^{\left(i\right)}$ is also termed “particles” in the PF.

In order to calculate the posterior distribution $p(x_{t}|y_{0:t})$ in the PF, a recursive approach is required. Specifically, this posterior distribution $p(x_{t}|y_{0:t})$ can be achieved by iteratively applying the following:

(8) $$p\left(x_{t}|y_{0:t}\right)\propto \int p\left(x_{t}|x_{t-1}\right)p\left(y_{t}|x_{t}\right)p\left(x_{t-1}|y_{0:t-1}\right)dx_{t-1}$$

Based on Equation (8), suppose we have the weighted sample set $\left(x_{t-1}^{\left(i\right)},W_{t-1}^{\left(i\right)}\right)$ generated from $p(x_{t-1}|y_{0:t-1})$. According to this assumption, a new sample $x_{t}^{\left(i\right)}$ is generated by randomly sampling from the state transition function $p(x_{t}|x_{t-1})$. The resulting set of weighted samples $\left\{\left(x_{t}^{\left(i\right)},x_{t-1}^{\left(i\right)},W_{t-1}^{\left(i\right)}\right)\right\}$ can approximate the joint distribution $p(x_{t},x_{t-1}|y_{0:t-1})$. The updated weights are defined by the following expression:

(9) $$W_{t}^{\left(i\right)}=p\left(y_{t}|x_{t}^{\left(i\right)}\right)W_{t-1}^{\left(i\right)},$$

With these updated weights, the following theorem holds:

**Theorem** **1.** *The weighted sample set ${\left\{\left(x_{t}^{\left(i\right)},x_{t-1}^{\left(i\right)},W_{t}^{\left(i\right)}\right)\right\}}_{i=1}^{N}$ approximates the joint distribution $p(x_{t},x_{t-1}|y_{0:t})$.*

**Proof.**  The weighted sample set $\left\{\left(x_{t}^{\left(i\right)},x_{t-1}^{\left(i\right)},W_{t-1}^{\left(i\right)}\right)\right\}$ provides an approximation to the joint distribution $p(x_{t},x_{t-1}|y_{0:t-1})$. Using the weight update in Equation (9), the weights for each particle $x_{t}^{\left(i\right)}$ are computed as follows:

(10) $$W_{t}^{\left(i\right)}=p\left(y_{t}|x_{t}^{\left(i\right)}\right)W_{t-1}^{\left(i\right)},$$

According to Bayesian theory, the joint distribution $p(x_{t},x_{t-1}|y_{0:t})$ can be expressed as

(11) $$p\left(x_{t},x_{t-1}|y_{0:t}\right)=\frac{p\left(y_{t}|x_{t},x_{t-1},y_{0:t-1}\right)p\left(x_{t},x_{t-1}|y_{0:t-1}\right)}{p(y_{t}|y_{0:t-1})}.$$

where $p(y_{t}|y_{0:t-1})$ is a normalization constant independent of $x_{t}$ and $x_{t-1}$. Additionally, according to Assumption 1, we have $p\left(y_{t}|x_{t},x_{t-1},y_{0:t-1}\right)=p\left(y_{t}|x_{t}\right)$. Then, Equation (11) can be rewritten as

(12) $$p\left(x_{t},x_{t-1}|y_{0:t}\right)\propto p\left(y_{t}|x_{t}\right)p\left(x_{t},x_{t-1}|y_{0:t-1}\right).$$

Since the weighted sample set ${\left\{\left(x_{t}^{\left(i\right)},x_{t-1}^{\left(i\right)},W_{t}^{\left(i\right)}\right)\right\}}_{i=1}^{N}$ approximates the joint posterior $p(x_{t},x_{t-1}|y_{0:t})$, this proof is complete.    □

**Figure 2:**
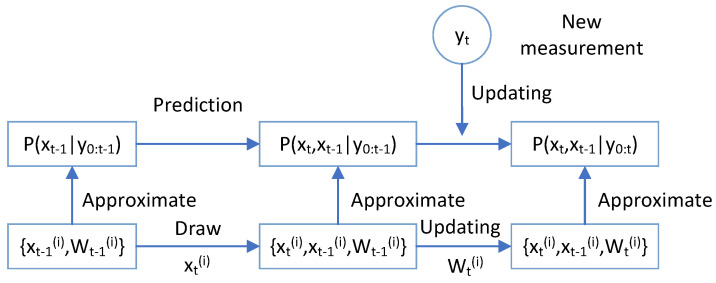
The PF approximates the joint posterior $p(x_{t},x_{t-1}|y_{0:t})$ process.

Based on the above two processes and Theorem 1, we can recursively obtain the weighted samples of $p(x_{t},x_{t-1}|y_{0:t})$ from $p(x_{t-1}|y_{0:t-1})$. Specifically, the PF approximates the joint posterior $p(x_{t},x_{t-1}|y_{0:t})$, which is shown in Figure 2.

Hence, we can obtain the weighted samples of posterior distribution $p(x_{t}|y_{0:t})$. From the above Theorem 1, it follows that the next theorem holds.

**Theorem** **2.** *The weighted sample set ${\left\{\left(x_{t}^{\left(i\right)},W_{t}^{\left(i\right)}\right)\right\}}_{i=1}^{N}$ provides an approximation to the posterior distribution $p(x_{t}|y_{0:t})$. Likewise, the weighted sample set ${\left\{\left(x_{t-1}^{\left(i\right)},W_{t}^{\left(i\right)}\right)\right\}}_{i=1}^{N}$ approximates the distribution $p(x_{t-1}|y_{0:t})$.*

By applying the above Theorem 2, we use the weighted sample set ${\left\{\left(x_{t}^{\left(i\right)},W_{t}^{\left(i\right)}\right)\right\}}_{i=1}^{N}$ to approximate the posterior distribution $p(x_{t}|y_{0:t})$. Additionally, the weighted samples for $p(x_{t-1}|y_{0:t})$ can be obtained using similar principles, addressing the one-step smoothing problem through a sampling approach. This leads to the proposal of a one-step particle smoother.

Based on the above description, we suppose that use the weighted sample set $\left(x_{t-1}^{\left(i\right)},W_{t}^{\left(i\right)}\right)$ approximates the distribution $p(x_{t-1}|y_{0:t})$. If we randomly generate new samples $x_{t}^{\left(i\right)}$ from the state transfer function $p(x_{t}|x_{t-1}^{\left(i\right)})$, then the new samples ${\left\{\left(x_{t}^{\left(i\right)},W_{t}^{\left(i\right)}\right)\right\}}_{i=1}^{N}$ also approximate the following distribution on $x_{t}$:

(13) $$\begin{array}{c} g_{t}\left(x_{t},y_{0:t}\right)=\int p\left(x_{t}|x_{t-1}\right)p\left(x_{t-1}|y_{0:t}\right)\;dx_{t-1}. \end{array}$$

**Figure 3:**
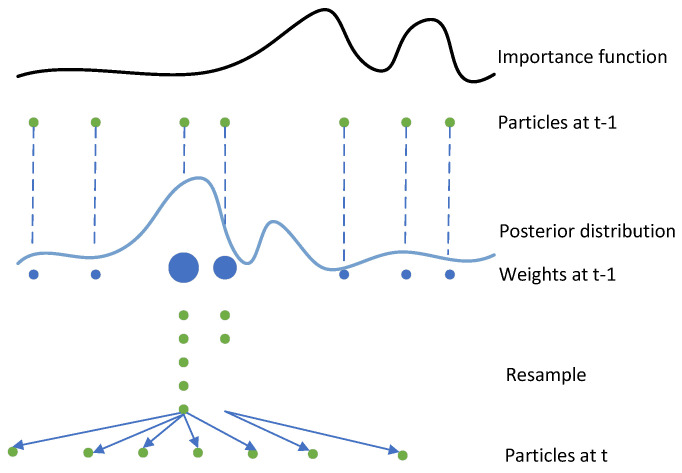
Particle degradation figure.

**Remark** **2.** *We use $g_{t}\left(x_{t},y_{0:t}\right)$ in Equation* (13) *as the importance function to generate weighted samples for the approximate posterior. This approach is called a particle filter based on one-step smoothing. The rationale for this choice is explained as follows. Based on Bayesian theory, upon receiving the measurement $y_{t}$, $p(x_{t}|y_{0:t-1})$ and $p(x_{t}|y_{0:t})$ represent the prior and posterior distributions, respectively. For the BPF, considering $p(x_{t}|y_{0:t})$ as the posterior distribution and $p(x_{t}|y_{0:t-1})$ as the importance function, it does not contain the current measurement information for the importance function, making it too different from the posterior distribution, which can easily lead to the particle degradation, which in turn affects the estimation accuracy. In order to understand the phenomenon of particle degradation more intuitively, we provide Figure 3, through which we clearly show the change of particles in the process of weight update and resampling. The primary reason for particle degradation is that the importance function significantly differs from the true posterior distribution. This mismatch causes particles to be sampled from low-probability regions of the posterior distribution, which leads to an uneven distribution of particle weights. Over the course of iterations, this imbalance causes the weight variance to increase progressively. As a result, most particles end up with very low weights, while only a few particles maintain high weights. During the resampling process, higher-weighted particles are repeatedly selected, while lower-weighted particles are discarded. Naturally, the sampling effectiveness of the BPF could be greatly enhanced if we find a way to incorporate the latest received measurements $y_{t}$ into the importance function rather than relying solely on the prior.*

## 4. A Novel Particle Filter Based on One-Step Smoothing for Nonlinear System with Random One-Step Delay and Missing Measurements

### 4.1. A Novel Particle Filter Based on One-Step Smoothing

In the BPF, the chosen importance function does not incorporate observation information, making it prone to particle degradation. Based on the previous discussion, selecting an appropriate importance function can effectively limit particle degradation. Therefore, in this subsection, we propose an iterative scheme to modify the one-step smoothing approach to incorporate the current measurement $y_{t}$ to obtain a better importance function. We denote the importance function as $g_{t}\left(x_{t},y_{0:t}\right)$. To implement this iterative scheme, a method for calculating the importance weights is required. Specifically, the importance weights are derived using the following theorem:

**Theorem** **3.** *The updated weights in the PF for a nonlinear system with random one-step delay and MM are expressed as*

(14) $$W_{t}^{\left(i\right)}\propto W_{t-1}^{\left(i\right)}\frac{p\left(y_{t}|x_{t}^{\left(i\right)},x_{t-1}^{\left(i\right)}\right)p\left(x_{t}^{\left(i\right)}|x_{t-1}^{\left(i\right)}\right)}{g_{t}\left(x_{t}^{\left(i\right)},y_{0:t}\right)}$$

*where*

(15) $$\begin{array}{cc} p(y_{t}|x_{t}^{\left(i\right)},x_{t-1}^{\left(i\right)})= & \;ap_{v_{t}}\left(y_{t}-h_{t}\left(x_{t}^{\left(i\right)}\right)\right)\\ \; & +\left(1-a\right)bp_{v_{t-1}}\left(y_{t}-h_{t-1}\left(x_{t-1}^{\left(i\right)}\right)\right)\\ \; & +\left(1-a\right)\left(1-b\right)p_{v_{t-1}}\left(y_{t}\right), \end{array}$$

(16) $$\begin{array}{cc} p(x_{t}|x_{t-1})\; & ={\left(2\pi \right)}^{-\frac{n}{2}}{\left|Q_{t-1}\right|}^{-\frac{1}{2}}\\ \; & \;\times \exp\left(-\frac{1}{2}{\left(x_{t}-f_{t-1}\left(x_{t-1}\right)\right)}^{T}{\left(Q_{t-1}\right)}^{-1}\left(x_{t}-f_{t-1}\left(x_{t-1}\right)\right)\right). \end{array}$$

*and $x_{t}^{\left(i\right)}$ is obtained by the importance function $g_{t}\left(x_{t},y_{0:t}\right)$. Here, $p_{v_{t}}\left(\cdot \right)$ and $p_{v_{t-1}}\left(\cdot \right)$ represent the PDF of the observation noises $v_{t}$ and $v_{t-1}$, respectively.*

**Proof.**  Based on Assumptions 1 and 2 and the theoretical framework of importance weight proposed in [20], when the improved importance function is denoted by $g_{t}\left(x_{t}^{\left(i\right)},y_{0:t}\right)$, its corresponding weight for nonlinear systems random one-step delay and MMs can be written in the form of Equation (14). Here, the detailed proof of $p(y_{t}|x_{t}^{\left(i\right)},x_{t-1}^{\left(i\right)})$ in Equation (15) can be found in [37]. Additionally, the state transfer function $p(x_{t}|x_{t-1})$ can be derived easily from the state Equation (1). This proof is complete.    □

To incorporate the information of the current measurement into the new importance function, the sample $x_{t}^{\left(i\right)}$ is chosen through a threshold determined by the distance between the measurements and the observation predicted value by using one-step smoothing. The use of the one-step smoothing with an iteration allows the sample $x_{t}^{\left(i\right)}$ to absorb the information of the current measurement $y_{t}$. Accordingly, the samples through the one-step smoothing scheme can be regarded as samples drawn from the importance function $g_{t}\left(x_{t},y_{0:t}\right)$. Based on Theorem 3, the one-step smoothing scheme with an iterative process is described as follows:

- Iteration initialization. Denote the one-step particle smoother $\left\{x_{t-1}^{\left(i\right)},W_{t}^{\left(i\right)}\right\}$ by $\left\{x_{t-1,0}^{\left(i\right)},W_{t,0}^{\left(i\right)}\right\}$ as the initial iteration, where i=1,…,N.
- Obtain sample $x_{t,j}^{\left(i\right)}$ from $p(x_{t}|x_{t-1})$.
- Compute $w_{t,j}^{\left(i\right)}$ by Equation (14).
- Denote $\left\{x_{t-1,j}^{\left(i\right)},W_{t,j}^{\left(i\right)}\right\}:=\left\{x_{t-1,j-1}^{\left(i\right)},W_{t,j}^{\left(i\right)}\right\}$.
- Stop the iteration when the specified stopping criterion is satisfied. The stopping condition is the threshold which is the distance between the measurements and the observations predicted values.

Denote $p_{j}\left(x_{t-1}|y_{0:t}\right)$ as the distribution of the *j*th iteration. The purpose of the iterative process is to progressively adjust the significant region of $p_{j}\left(x_{t-1}|y_{0:t}\right)$ to ensure that the state $x_{t}$ propagated from any sample within this region has an increased likelihood of being in the high-likelihood region of $p(y_{t}|x_{t}^{\left(i\right)},x_{t-1}^{\left(i\right)})$. Through this iterative process, we can become closer to the importance function of the posterior [22].

The stopping rules used are as follows. First, a small positive number ρ is chosen as the threshold. Then, the observation predicted value $y_{pr}^{\left(i\right)}$ of the particle $x_{t}^{\left(i\right)}$ is computed via the following theorems:

**Theorem** **4.** *Based on Equations* (2) *and* (3)*, the observation predicted value $y_{pr}^{\left(i\right)}$ can be described as follows:*

(17) $$y_{pr}^{\left(i\right)}=ah\left(x_{t}^{\left(i\right)}\right)+\left(1-a\right)b\left(h\left(x_{t-1}^{\left(i\right)}\right)+v_{t-1}\right).$$

**Proof.**  (18) $$\begin{array}{cc} y_{pr} & =E(y_{t}|y_{0:t-1})\\ \; & =E(\lambda_{t}z_{t}+\left(1-\lambda_{t}\right)\xi_{t}z_{t-1}+\left(1-\lambda_{t}\right)\left(1-\xi_{t}\right)v_{t}|y_{0:t-1})\\ \; & =aE\left(z_{t}|y_{0:t-1}\right)+\left(1-a\right)bE\left(z_{t-1}|y_{0:t-1}\right) \end{array}$$

(19) $$\begin{array}{cc} \; & =aE\left(h\left(x_{t}\right)|y_{0:t-1}\right)+\left(1-a\right)bE\left(h\left(x_{t-1}\right)+v_{t-1}\right)\left|y_{0:t-1}\right)\\ \; & =aE\left(h\left(x_{t}\right)|y_{0:t-1}\right)+\left(1-a\right)b\left(E\left(h\left(x_{t-1}\right)|y_{0:t-1}\right)+E\left(v_{t-1}|y_{0:t-1}\right)\right)\\ \; & =ah\left(x_{t}\right)+\left(1-a\right)b\left(h\left(x_{t-1}\right)+v_{t-1}\right) \end{array}$$

This proof is complete.    □

Secondly, calculate the distance between the observation predicted value $y_{pr}^{\left(i\right)}$ and the current measurement $y_{t}$ through

(20) $$d_{t}^{\left(i\right)}=\left|\right|y_{t}-y_{pr}^{\left(i\right)}\left|\right|.$$

Finally, stop the iterations if $d_{t}^{\left(i\right)}<\rho$.

**Figure 4:**
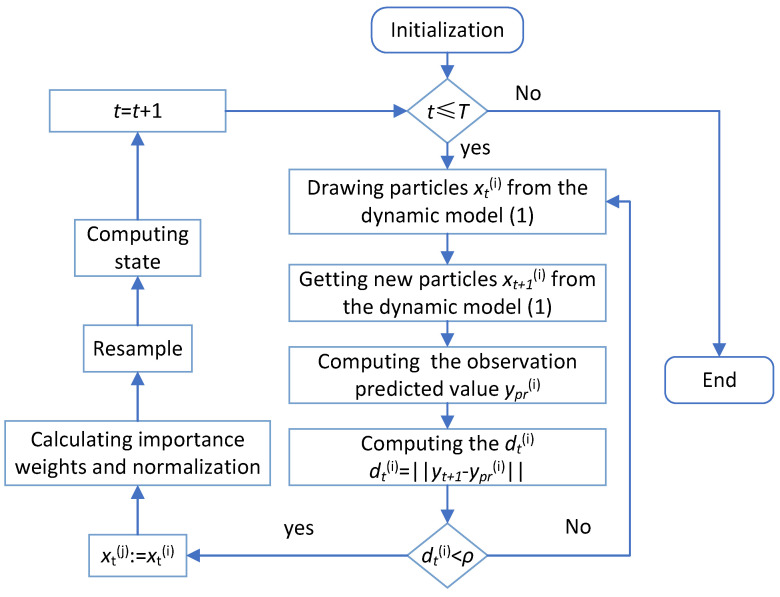
One-step smoothing scheme with an iterative process.

This stopping rule stems from the recognition that if the obtained importance function is near the posterior distribution, then the observation predicted value will be close to the real measurement. For a more intuitive understanding of the one-step smoothing scheme and its iterative process, we provide the following Figure 4.

The proposed PF using one-step smoothing with iteration employs the current measurement $y_{t}$ to judge the difference between the posterior and the importance function derived through the iterations. If the distance $d_{t}^{\left(i\right)}$ does not exceed a set threshold ρ, then we receive sample $x_{t}^{\left(i\right)}$; otherwise, we reject sample $x_{t}^{\left(i\right)}$, continue to draw sample $x_{t}^{\left(i\right)}$ instead and repeat the above operation until we obtain *N* samples $x_{t}^{\left(i\right)}$. The theory stems from the realization that if the prediction of the sample observation is close to the true measurement, the obtained importance function is close to the posterior distribution. Then, the weights for each sample $x_{t}^{\left(i\right)}$ are obtained by Equation (15). Resample the particle set $\left\{x_{t}^{\left(i\right)},{\bar{W}}_{t}^{\left(i\right)}\right\}$.

For nonlinear systems with random one-step delay and MMs, the proposed PF employs one-step smoothing through an iterative process to incorporate the current measurement into the importance function, thereby obtaining a better importance function and improving the state estimation accuracy.

### 4.2. Algorithm

According to Theorem 3 and the one-step smoothing technique by iteration, an algorithm can be developed to acquire a novel PF for the nonlinear system with random one-step delay and MMs. We design this new PF as the following Algorithm 1.

**Algorithm 1** A novel particle filter with random one-step delay and MM.
**Step I. Initialization:**
Draw state particles ${\left\{x_{0}^{\left(i\right)}\right\}}_{i=1}^{N}$ from prior $p(x_{0})$.The weight of all particles is $\frac{1}{N}$For t=1,2,…
**Step II. Repeat the following 1–9 steps:**
1. Obtain new sample $x_{t}^{\left(i\right)}$ by dynamic model (1).$x_{t}^{\left(i\right)}=f\left(x_{t-1}^{\left(i\right)}\right)+w_{t-1}^{\left(i\right)}$.2. Obtain new sample $x_{t+1}^{\left(i\right)}$ by dynamic model (1).$x_{t+1}^{\left(i\right)}=f\left(x_{t}^{\left(i\right)}\right)+w_{t}^{\left(i\right)}$.3. Calculate the observation predicted value $y_{pr}^{\left(i\right)}$ by Equation (18).4. Calculate the the distance between the measurements and the observations’ predicted values.$d_{t}^{\left(i\right)}=\left|\right|y_{t+1}-y_{pr}^{\left(i\right)}\left|\right|$.5. Judge whether the distances are larger
than the threshold value.For j=1:NIf $d_{t}^{\left(i\right)}<\rho$   $x_{t}^{\left(j\right)}=x_{t}^{\left(i\right)}$else   Return to the step 1–5.6. Evaluate importance weights by Equation (14).7. Normalize importance weights:   ${\bar{W}}_{t}^{\left(i\right)}=\frac{W_{t}^{\left(i\right)}}{\sum_{i=1}^{N}W_{t}^{\left(i\right)}}$.8. Resample, i.e.,   ${\left\{{\tilde{x}}_{t}^{\left(i\right)},{\tilde{W}}_{t}^{\left(i\right)}=\frac{1}{N}\right\}}_{i=1}^{N}=$ Resample ${\left\{x_{t}^{\left(i\right)},{\bar{W}}_{t}^{\left(i\right)}\right\}}_{i=1}^{N}$.9. Compute the state estimation,   ${\hat{x}}_{t}=\sum_{i=1}^{N}{\tilde{x}}_{t}^{\left(i\right)}{\tilde{W}}_{t}^{\left(i\right)}$.The end.

## 5. Simulation

To justify the effectiveness of the proposed novel particle filter (NPF), a simulation experiment is provided in this section to compare it with the existing bootstrap particle filter (BPF) for nonlinear systems with random one-step delay and MMs.

Consider dynamic systems with state space formalisms that can be described in the following:

(21) $$\begin{array}{cc} \; & x_{t+1}=\left[\begin{array}{c} \sin\left(x_{1,t}\right)x_{1,t}+\cos\left(2t\right)x_{2,t}\\ \cos\left(x_{2,t}\right)x_{2,t}+0.75x_{2,t} \end{array}\right]+w_{t}, \end{array}$$

(22) $$\begin{array}{cc} \; & z_{t}=\left[\begin{array}{c} \sin\left(2t\right)x_{1,t}+x_{1,t}x_{2,t}\\ \cos\left(2x_{2,t}\right)x_{1,t}+\sin\left(t\right)x_{2,t} \end{array}\right]+v_{t}, \end{array}$$

(23) $$\begin{array}{cc} \; & y_{t}=\lambda_{t}z_{t}+\left(1-\lambda_{t}\right)\xi_{t}z_{t-1}+\left(1-\lambda_{t}\right)\left(1-\xi_{t}\right)v_{t}. \end{array}$$

where the state vector $x_{t}={\left[x_{1,t},x_{2,t}\right]}^{T}$, the system noise $w_{t}={\left[w_{1,t},andw_{2,t}\right]}^{T}$, the observation noise $v_{t}={\left[v_{1,t},v_{2,t}\right]}^{T}$, $w_{t}$ and $v_{t}$ are mutually independent Gaussian white noise that satisfy these properties: $w_{t}∼N\left(0,Q_{t}\right)$, $v_{t}∼N\left(0,R_{t}\right)$.

The random variables $\lambda_{t}$ and $\xi_{t}$, representing random one-step delay and MMs, follow a Bernoulli distribution with the following statistical characteristics:

$$\begin{array}{cc} P(\lambda_{t}=1) & =E\{\lambda_{t}\}=a,\\ P(\lambda_{t}=0) & =1-E\{\lambda_{t}\}=1-a,\\ P(\xi_{t}=1) & =E\{\xi_{t}\}=b,\\ P(\xi_{t}=0) & =1-E\{\xi_{t}\}=1-b, \end{array}$$

where a and b are known constants in the interval [0, 1].

Let $x_{0}={\left[2,2\right]}^{T}$, ${\hat{x}}_{0|0}={\left[2,2\right]}^{T}$, $Q_{t}=0.2I_{2}$, $R_{t}=0.005I_{2}$, $P_{0|0}=2I_{2}$, a=0.8, b=0.8.

**Figure 5:**
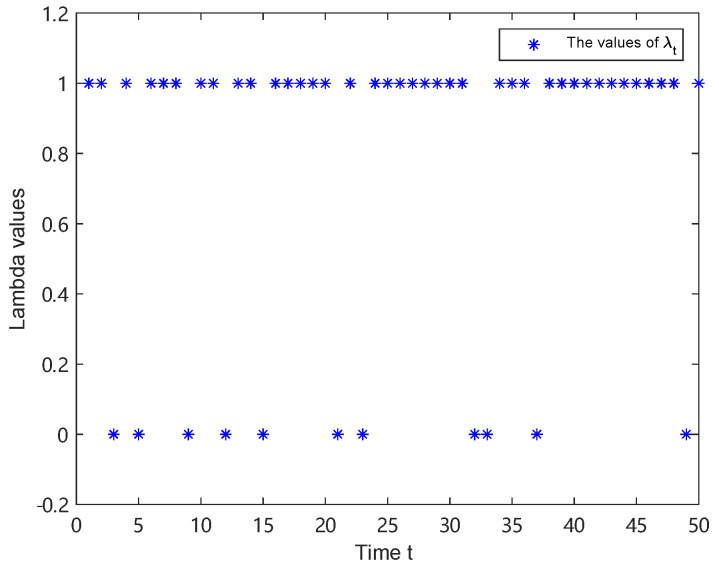
The value of $\lambda_{t}$.

**Figure 6:**
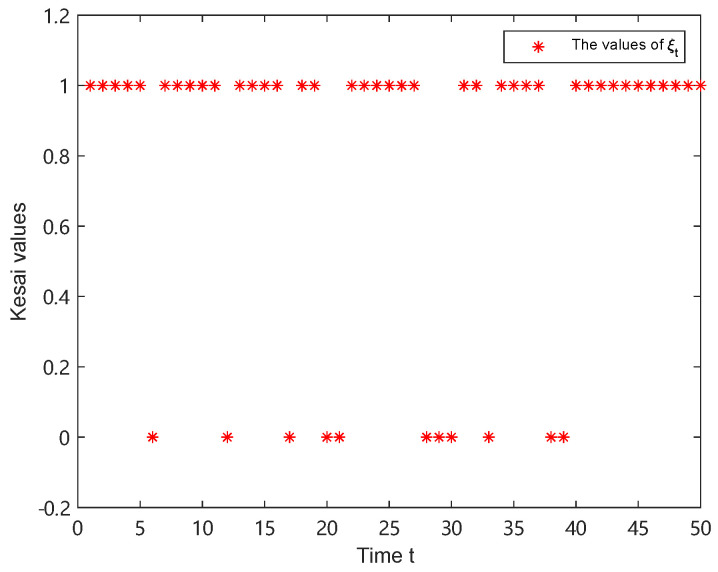
The value of $\xi_{t}$.

The experiment was set up for 50 time steps, and the simulation used 100 particles. The trajectories of $\lambda_{t}$ and $\xi_{t}$ are shown in Figure 5 and Figure 6, respectively. It follows that the appearance of one-step delay and MMs is random.

**Figure 7:**
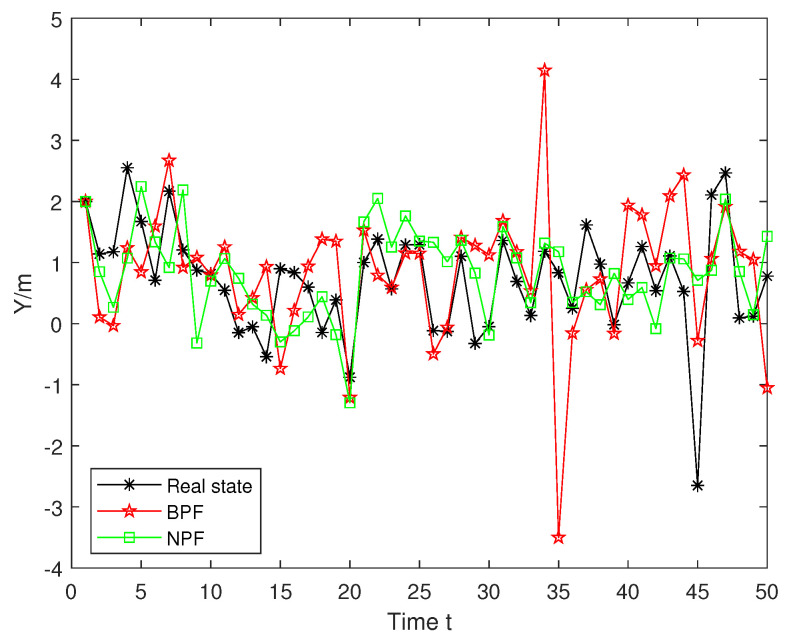
True and estimated trajectories for $x_{1}$.

To evaluate the performance of the proposed NPF and BPF, Figure 7 compares the true trajectory of $x_{1}$ with the trajectories estimated using the BPF and NPF. The NPF demonstrates greater stability over the entire time range with estimated trajectories closely aligning with the true trajectory. In contrast, the BPF exhibits noticeable deviations from the true trajectory, particularly at time steps 33–35. These results highlight the better performance of the NPF compared to the BPF.

**Figure 8:**
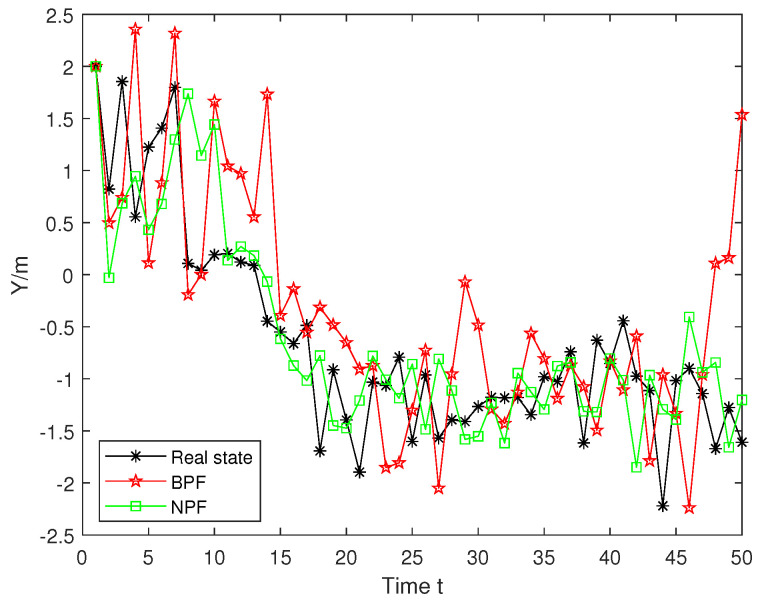
True and estimated trajectories for $x_{2}$.

Likewise, we evaluate the true trajectory of $x_{2}$ with the trajectories estimated by the BPF and NPF, as shown in Figure 8. The NPF demonstrates greater stability throughout the time range with its estimated trajectories closely following the true trajectory. In contrast, the BPF exhibits significant deviations from the true trajectory at most time steps. From these two trajectory figures, we can see that the NPF has a better advantage compared to the BPF.

The above trajectory figures above illustrate the estimates obtained from the two algorithms over time. However, these estimates alone are not sufficient to assess the overall accuracy and stability of these algorithms. To offer a more comprehensive evaluation, we introduce two performance metrics: estimation accuracy and computational complexity.

First, we introduce the root mean square error (RMSE) to assess the estimation accuracy of the BPF and NPF, i.e.,

(24) $$RMSE_{x}\left(t\right)=\sqrt{\frac{1}{MC}\sum_{n=1}^{MC}{\left({\hat{x}}_{t,n}-x_{t,n}\right)}^{2}}.$$

**Figure 9:**
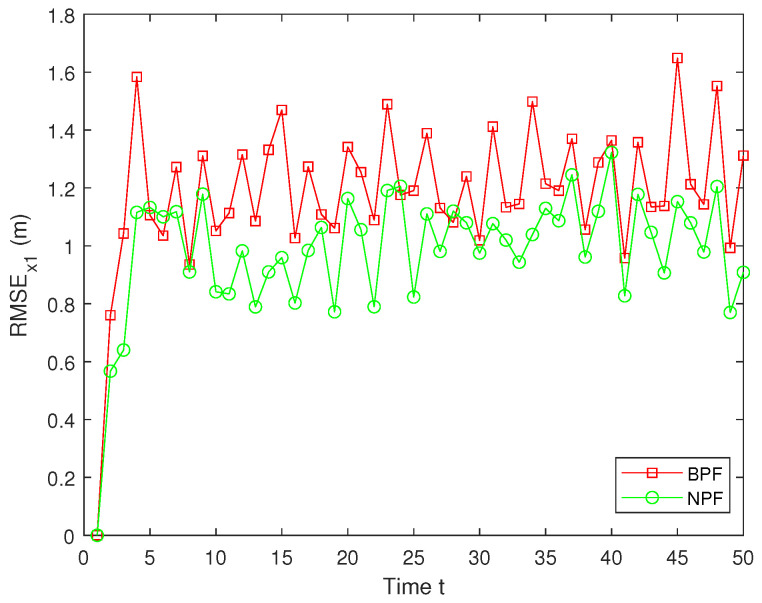
RMSE of $x_{1}$.

Here, ${\hat{x}}_{t}$ represents the estimated value of $x_{t}$. To guarantee the validity of the results, MC=100 independent Monte Carlo simulations are conducted, and the simulation outcomes are derived from these experiments. The RMSE values of the estimation results for $x_{1}$ using the BPF and NPF are shown in Figure 9. The experimental results demonstrate that the NPF consistently exhibits smaller errors and higher accuracy over time. In contrast, the BPF shows larger errors at all time steps with notable error peaks occurring at several points during the simulation. These figures highlight the superior performance of the NPF compared to the BPF.

**Figure 10:**
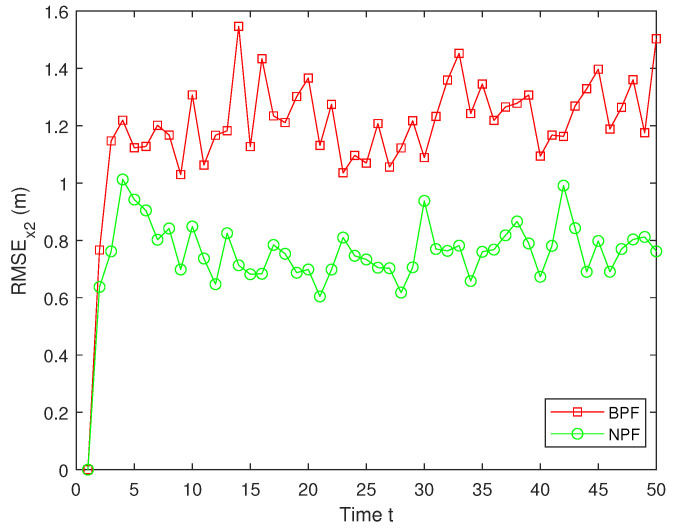
RMSE of $x_{2}$.

The RMSEs of the estimation results for $x_{2}$ by the BPF and NPF are shown in Figure 10. The experimental results show that the NPF consistently maintains a small error throughout the time, exhibiting superior estimation accuracy and stability. In contrast, the estimation errors for the BPF are significantly larger, with large fluctuations at several time steps, indicating their limitations in dealing with nonlinear systems (1)–(3). Furthermore, the average RMSE (ARMSE) of two PFs for $x_{1}$ and $x_{2}$ are shown in Table 1. As can be seen from it, the NPF is significantly more accurate than the BPF, with the NPF performing the best in terms of average filtering accuracy, outperforming the existing BPF algorithm.

Computational complexity has always been a concern in practical applications of filtering. Therefore, we ran 100 Monte Carlo simulations of the two PFs and recorded their average computation times in Table 1. It is shown that the NPF has a longer computational time than the BPF. The computational efficiency of the NPF is generally lower because a large amount of computing resources are used to select the sample $x_{t}^{\left(i\right)}$. Despite the increase in computation time, this increase can significantly improve the accuracy of the filters.

**Figure 11:**
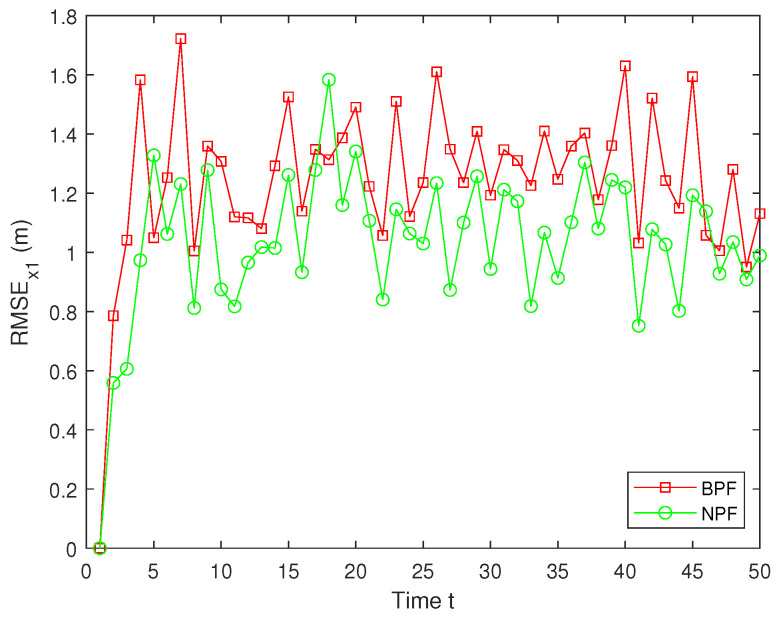
RMSE of $x_{1}$ (a = 0.8, b = 0.6).

**Figure 12:**
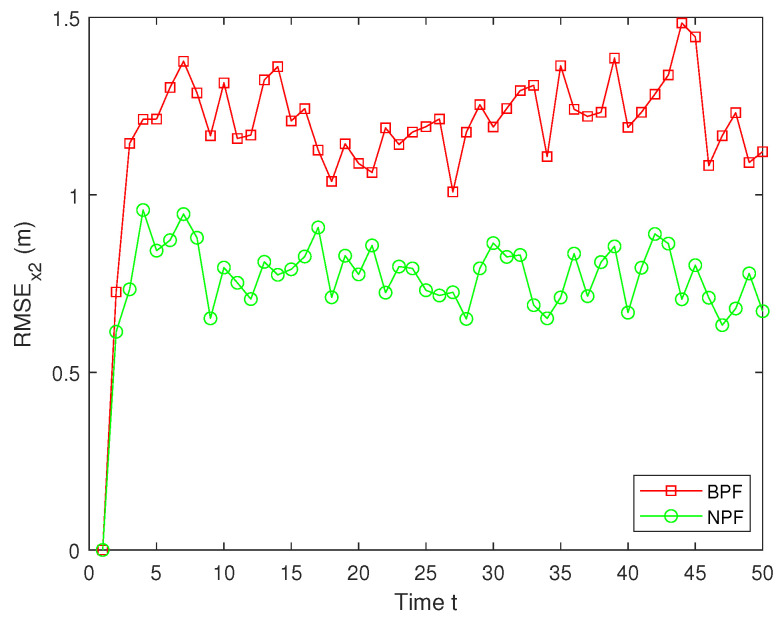
RMSE of $x_{2}$ (a = 0.8, b = 0.6).

**Figure 13:**
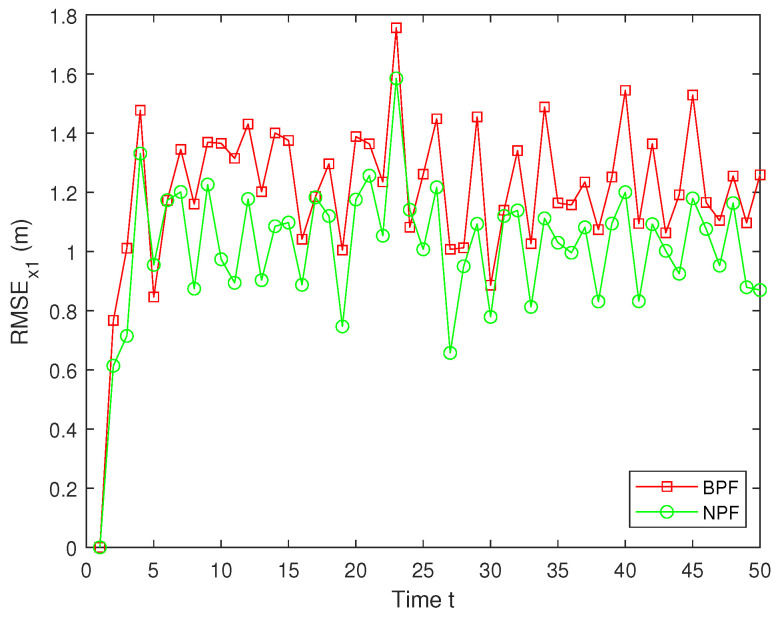
RMSE of $x_{1}$ (a = 0.8, b = 0.7).

**Figure 14:**
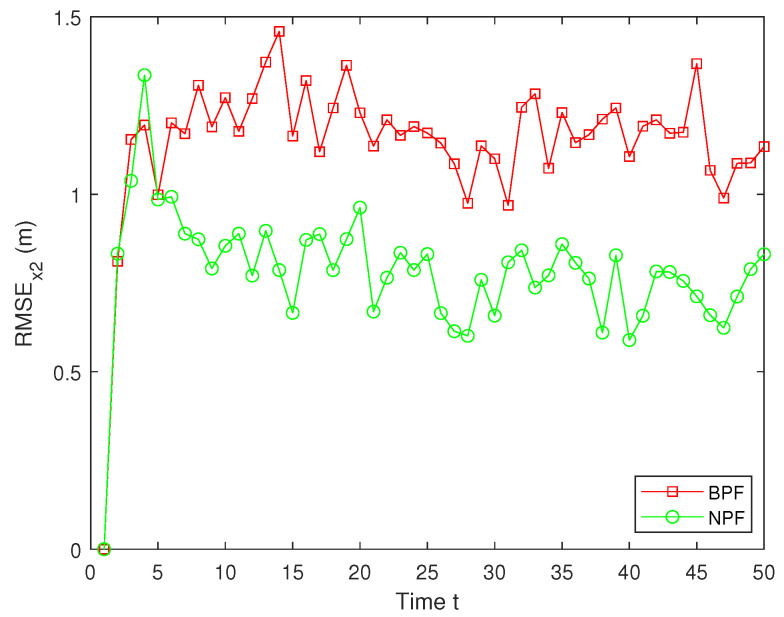
RMSE of $x_{2}$ (a = 0.8, b = 0.7).

**Figure 15:**
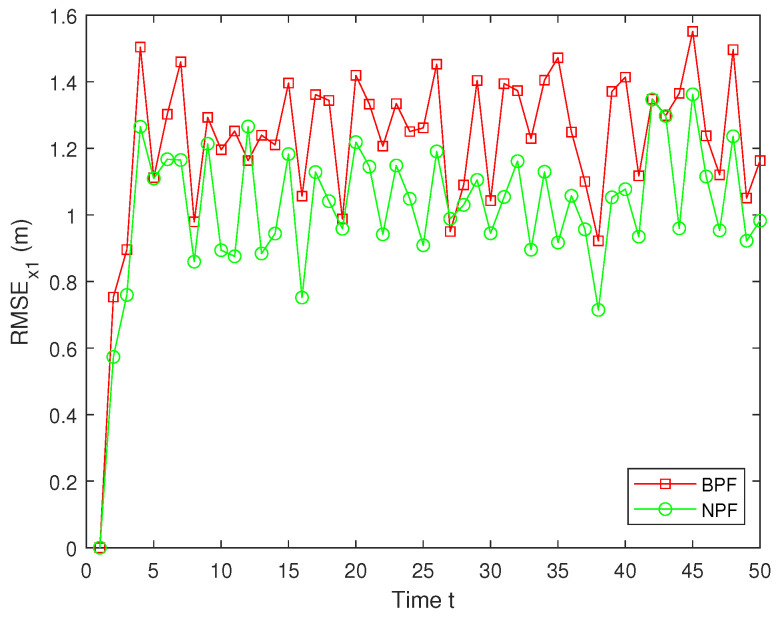
RMSE of $x_{1}$ (a = 0.8, b = 0.9).

**Figure 16:**
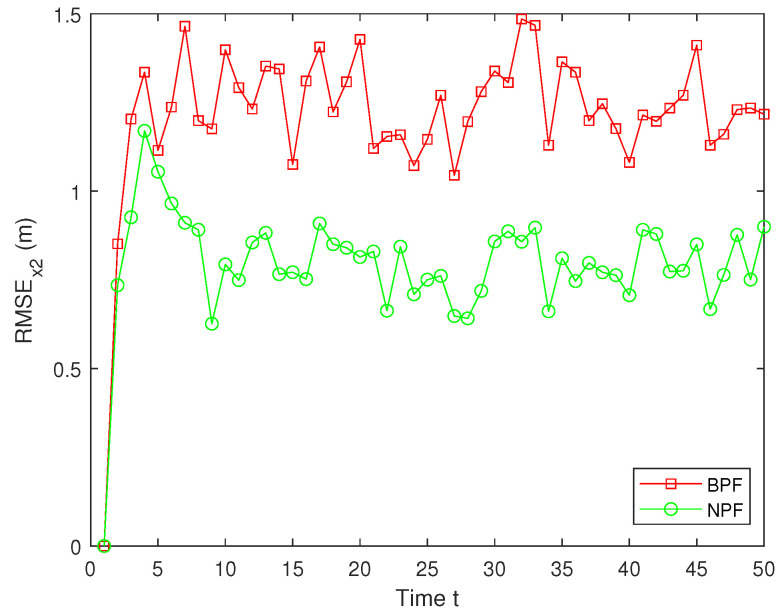
RMSE of $x_{2}$ (a = 0.8, b = 0.9).

**Figure 17:**
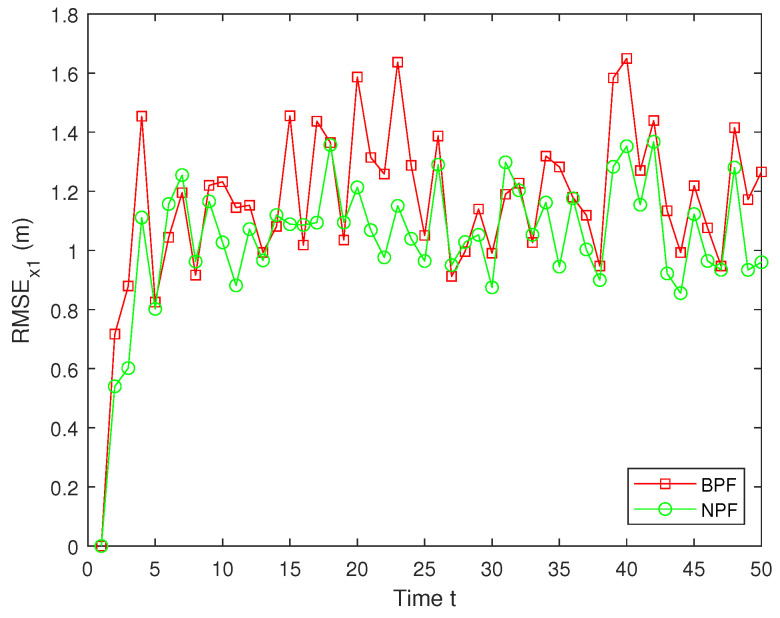
RMSE of $x_{1}$ (a = 0.5, b = 0.7).

**Figure 18:**
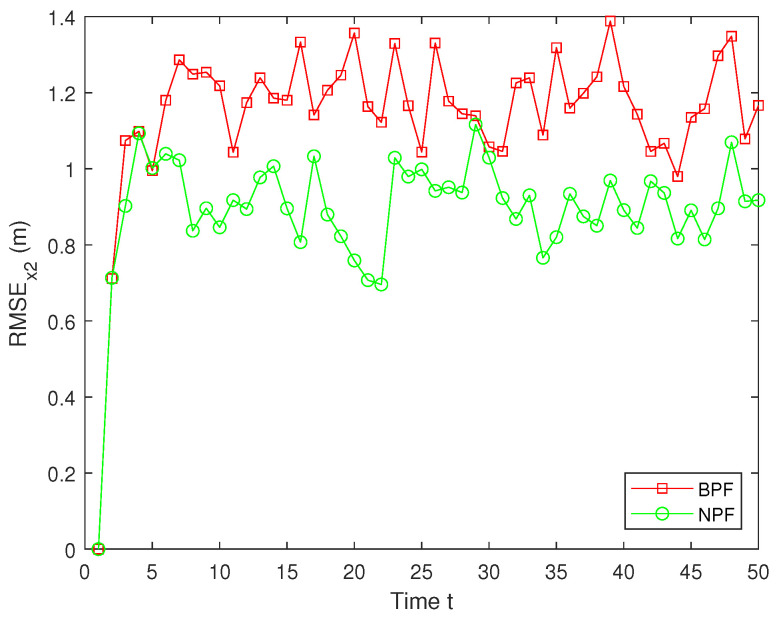
RMSE of $x_{2}$ (a = 0.5, b = 0.7).

**Figure 19:**
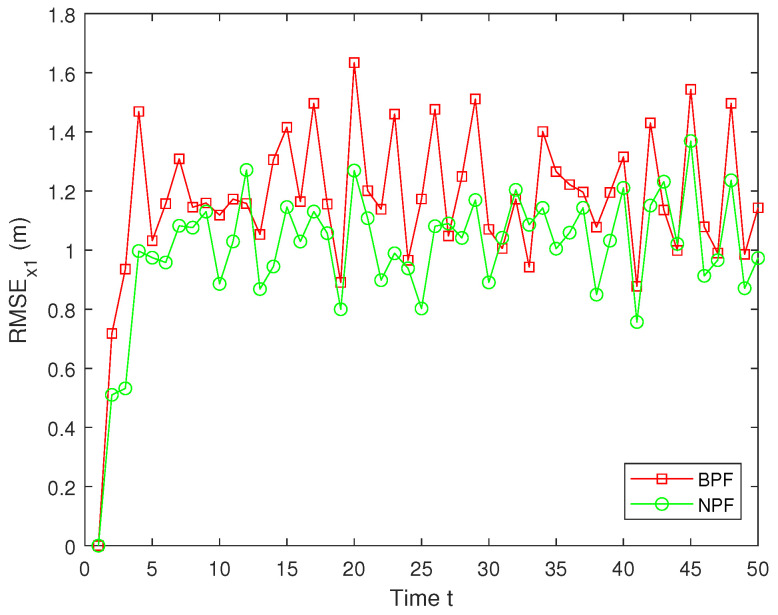
RMSE of $x_{1}$ (a = 0.7, b = 0.7).

**Figure 20:**
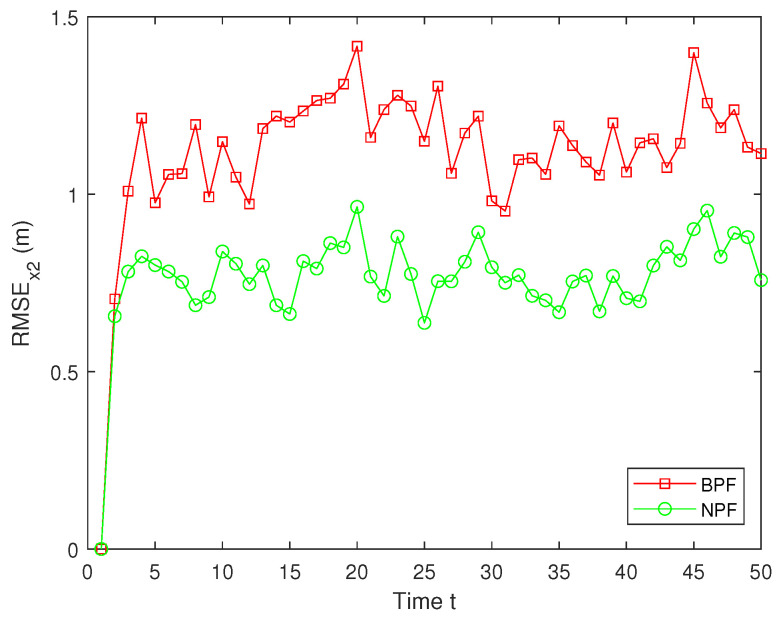
RMSE of $x_{2}$ (a = 0.7, b = 0.7).

**Figure 21:**
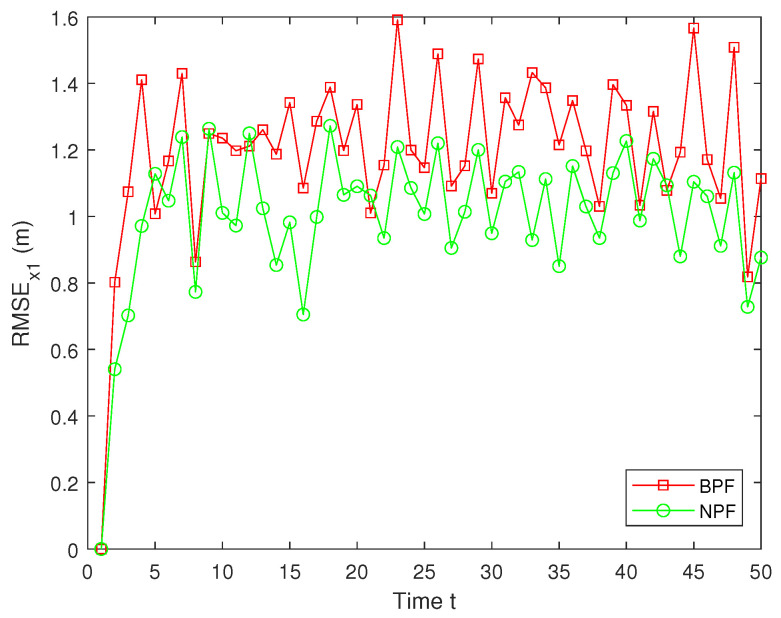
RMSE of $x_{1}$ (a = 0.85, b = 0.7).

**Figure 22:**
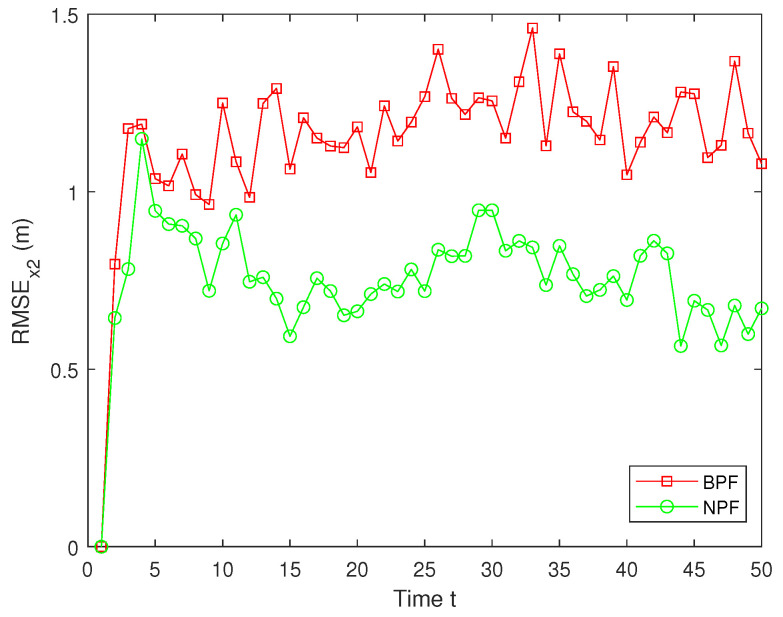
RMSE of $x_{2}$ (a = 0.85, b = 0.7).

In real systems, such as wireless sensor networks and control systems, one-step delay and MMs are common problems due to bandwidth limitations, network congestion, or sensor failures. However, both the one-step delay and missing measurements rate are uncertain. These factors can significantly affect the accuracy and stability of real-time data processing and decision-making processes. Therefore, in this paper, we analyze and compare different one-step delay rates and missing measurements rates in order to compare the accuracy and stability of the BPF and NPF algorithms in more depth. In the following, we selected the combinations where a=0.8 with b=0.6,0.7,0.9, and we analyzed the performance of the BPF and NPF in comparison. The corresponding simulation results of RMSE for $x_{1}$ and $x_{2}$ are presented in Figure 11, Figure 12, Figure 13, Figure 14, Figure 15 and Figure 16. To further evaluate the performance of the proposed NPF under the conditions of random one-step delay and MMs, we conducted experiments with b=0.7 and a=0.5,0.7,0.85. The simulation results, shown in Figure 17, Figure 18, Figure 19, Figure 20, Figure 21 and Figure 22, demonstrate that the NPF achieves smaller errors than the BPF at almost every time step across different scenarios based on 100 Monte Carlo simulations. These results highlight the effectiveness of the NPF in limiting particle degradation and improving estimation accuracy in complex dynamic systems.

## 6. Conclusions

This paper proposes a novel particle filter for nonlinear systems with random one-step delays and missing measurements. Such challenges often arise in networked control systems, where communication constraints, sensor failures, or network congestion can lead to random delays and missing data, significantly impacting the system’s performance. Two random variables obeying a Bernoulli distribution represent random one-step delay and missing measurements. This filter applies one-step smoothing by iteratively incorporating the current measurements into the importance function, resulting in a new importance function. Based on this importance function, an explicit expression for the corresponding weights is provided, leading to a novel particle filter algorithm for nonlinear systems with random one-step delay and missing measurements. Finally, the simulation examples show that the proposed novel particle filter has higher estimation accuracy and effectively limits particle degradation compared to bootstrap particle filters. For future work, alternative methods can be explored for constructing the importance function of particle filters to further enhance sampling efficiency, estimation accuracy, and computational efficiency in nonlinear systems with random one-step delay and missing measurements.

## Figures and Tables

**Table 1:** Average RMSEs and average computation Time.

Filters	RMSEs of $x_{1}$	RMSEs of $x_{2}$	Time (s)
BPF	1.2263	1.1190	0.0117
NPF	1.0409	0.7790	0.0341

## Data Availability

Data are contained within the article.
